# Allogeneic mesenchymal stem cells as induction therapy are safe and feasible in renal allografts: pilot results of a multicenter randomized controlled trial

**DOI:** 10.1186/s12967-018-1422-x

**Published:** 2018-03-07

**Authors:** Qipeng Sun, Zhengyu Huang, Fei Han, Ming Zhao, Ronghua Cao, Daqiang Zhao, Liangqing Hong, Ning Na, Heng Li, Bin Miao, Jianmin Hu, Fanhang Meng, Yanwen Peng, Qiquan Sun

**Affiliations:** 1 Department of Renal Transplantation, The Third Affiliated Hospital, Sun Yat-sen University, Kaichuang Road 2693, Huangpu District, Guangzhou, 510530 People’s Republic of China; 2 Department of Renal Transplantation, Zhujiang Hospital, Southern Medical University, Gongye Road 253, Guangzhou, 510280 People’s Republic of China; 3 Department of Renal Transplantation, The Second Affiliated Hospital, Guangzhou Traditional Chinese Medicine University, Inner Ring Road 55, University City, Guangzhou, 510280 People’s Republic of China; 4 Cell-gene Therapy Translational Medicine Research Center, The Third Affiliated Hospital, Sun Yat-sen University, Tianhe Road 600, Guangzhou, 510630 People’s Republic of China

**Keywords:** Mesenchymal stem cell, MSC, Renal transplantation, Delayed graft function, DGF, Acute rejection

## Abstract

**Background:**

Kidneys from deceased donors are being used to meet the growing need for grafts. However, delayed graft function (DGF) and acute rejection incidences are high, leading to adverse effects on graft outcomes. Optimal induction intervention should include both renal structure injury repair and immune response suppression. Mesenchymal stem cells (MSCs) with potent anti-inflammatory, regenerative, and immune-modulatory properties are considered a candidate to prevent DGF and acute rejection in renal transplantation. Thus, this prospective multicenter paired study aimed to assess the clinical value of allogeneic MSCs as induction therapy to prevent both DGF and acute rejection in deceased donor renal transplantation.

**Methods:**

Forty-two renal allograft recipients were recruited and divided into trial and control groups. The trial group (21 cases) received 2 × 10^6^/kg human umbilical-cord-derived MSCs (UC-MSCs) via the peripheral vein before renal transplantation, and 5 × 10^6^ cells via the renal artery during the surgical procedure. All recipients received standard induction therapy. Incidences of DGF and biopsy-proven acute rejection were recorded postoperatively and severe postoperative complications were assessed. Graft and recipient survivals were also evaluated.

**Results:**

Treatment with UC-MSCs achieved comparable graft and recipient survivals with non-MSC treatment (P = 0.97 and 0.15, respectively). No increase in postoperative complications, including DGF and acute rejection, were observed (incidence of DGF: 9.5% in the MSC group versus 33.3% in the non-MSC group, P = 0.13; Incidence of acute rejection: 14.3% versus 4.8%, P = 0.61). Equal postoperative estimated glomerular filtration rates were found between the two groups (P = 0.88). All patients tolerated the MSCs infusion without adverse clinical effects. Additionally, a multiprobe fluorescence in situ hybridization assay revealed that UC-MSCs administered via the renal artery were absent from the recipient’s biopsy sample.

**Conclusions:**

Umbilical-cord-derived MSCs can be used as clinically feasible and safe induction therapy. Adequate timing and frequency of UC-MSCs administration may have a significant effect on graft and recipient outcomes.

*Trial registration* NCT02490020. Registered on June 29 2015

## Background

Renal transplantation is currently the preferred treatment for patients with end-stage renal failure [1]. To address the widening gap between the growing need for renal transplantation and the availability of organs from brain-dead donors, the use of kidneys from donors after circulatory death has increased and become the major source of transplants in several countries [2]. However, during deceased donor renal transplantation, prolonged ischemic time from donor harvest to kidney reperfusion in the recipients resulted in higher incidences of delayed graft function (DGF) and acute rejection, which has adverse effects on grafts outcomes [3–6]. For renal allografts, DGF is primarily a consequence of pre-transplant injury and immune responses after reperfusion, whereas acute rejection is related to T cell clonal expansion and differentiation of effector cells during donor kidney injury [7]. Hence, the optimal therapy to prevent both DGF and acute rejection for renal transplantation using deceased donors should be able to repair renal structure injury and suppress immune response simultaneously. However, no current induction therapies possessed such capacity.

Mesenchymal stem cells (MSCs) present significant anti-inflammatory, tissue repair, and immune-modulatory properties, and could be used in a novel cell-based approach in renal transplantation. Effects of MSCs have been explored in several preclinical models of acute kidney injury (AKI) [8]. MSCs could attenuate the process of interstitial fibrosis/tubular atrophy, reduce macrophage infiltration and inflammatory cytokine expression, and increase anti-inflammatory factors in a rat kidney allograft model [9, 10]. Although initial trials showed safety and feasibility of MSCs treatment in renal transplantation, few data on its capacity to prevent both DGF and acute rejection have been reported, and the disadvantages associated with uncertainty of influencing factors, timing, dosage, route of administration, and frequency of treatment had negative effects on the clinical application of MSCs [11–14].

Thus, we conducted a multicenter randomized controlled trial of MSCs to clarify the clinical value of allogeneic MSCs, i.e., human umbilical-cord-derived MSCs (UC-MSCs), as induction therapy to prevent both DGF and acute rejection in deceased donor renal transplantation.

## Methods

### Study design

This is a prospective multicenter paired study including three kidney transplant institutions (The Third Affiliated Hospital of Sun Yat-sen University, Zhujiang Hospital of Southern Medical University, and The Second Affiliated Hospital of Guangzhou Traditional Chinese Medicine University). The study protocol was approved by the three institutions’ Ethics Committee of Human Study, which was established according to the Operational Guidelines for Ethics Committees that Review Biomedical Research developed by the World Health Organization (WHO) [15].

No organs from executed prisoners were used in the study, and the kidneys from all donors were procured in accordance with the WHO principles, Declaration of Helsinki, and Istanbul Declaration [16, 17].

Donors were selected based on confirmed patient identity, age ≤ 65 years, no history of kidney disease, drug abuse or uncontrollable psychotic symptoms, no active infection including HIV, bacteria or fungus, no history of uncontrolled hypertension, diabetes mellitus with complications, no history of malignant melanoma, metastatic or incurable malignancy [18].

Organ donation and recovery was facilitated by organ procurement organizations in the three kidney transplant institutions, which were established by the National Health and Family Planning Commission of China. Before procurement, written informed consent was obtained from the donor’s immediate family, who agreed to withdraw life support and donate the kidney. The obtained consent was provided to the Organ Donation Committee, which supervised the donation process. Organ procurement and management was strictly processed according to the national guidelines for donation after cardiac death in China [18].

### Participants

**Fig. 1:**
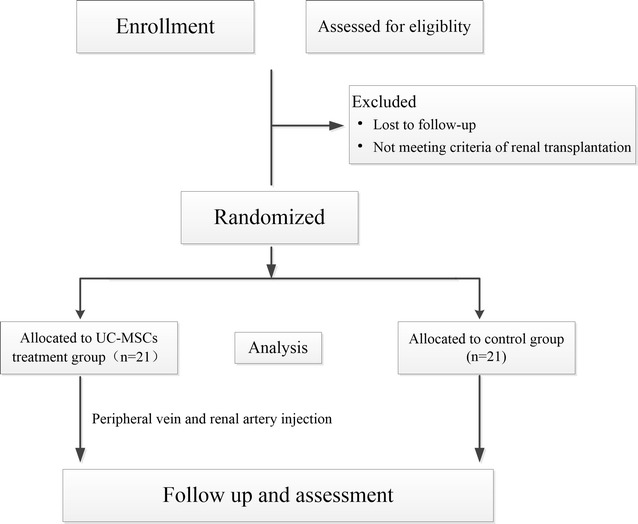
Study design and protocol for UC-MSCs administration in renal transplantation to prevent DGF and acute rejection

Based on preliminary studies [3, 19], 15 patients per arm should be required to achieve a power of 90% with a two-sided significance level of P < 0.05. To account for possible dropouts, the target number of patients was, therefore, set at 20 per arm. In the pilot study, 42 participants who received graft donations from the same donors from January 2016 to December 2016 were recruited and divided into two groups randomly. The participants were assigned to either the UC-MSCs treatment group or the control group in a 1:1 ratio using a block randomization method. A randomization list has been pregenerated. The participants were blinded to the treatment group throughout the study. The trial group simultaneously received UC-MSCs via the peripheral vein before operation and via the renal artery during operation. All the participants were recruited from the three transplant units. Written informed consent to participate in this study was obtained from the participants (Fig. 1).

### Endpoints

The primary endpoints of this study included DGF in 1 week post transplantation, biopsy proven acute rejection (BPAR) in 1 year. The secondary endpoints were severe opportunistic infections related with opportunistic infection as well as pulmonary and urinary tract infection, and other donor specific immune response in 1 year postoperatively.

### Immunosuppressive regimen

Antithymocyte globulin (50 mg/day) and methylprednisolone (500 mg/day) were continuously administered intravenously during the first 3 postoperative days as induction therapy. Maintenance immunosuppressive regimens consisted of a calcineurin inhibitor, mycophenolate mofetil, and prednisone. Mycophenolate mofetil, which was initiated immediately after transplantation, was maintained at a daily dose of 1.0–1.5 g. Tacrolimus or cyclosporine was started on days 2–4 at 0.1–0.15 or 6–8 mg/kg/day, respectively, based on the level of recovery of renal graft function. The immunosuppressive regimens were adjusted to achieve the target therapeutic trough levels in the peripheral blood (5–8 ng/ml for tacrolimus, and 130–150 ng/ml for cyclosporine). Oral administration of prednisone, which was initiated at 30 mg/day on day 4 following transplantation, was reduced by 5 mg/week until a maintenance dose of 10–15 mg/day.

### Umbilical cord blood units

The UC-MSCs used in this study were isolated after birth, with written consent from the parents, and a total volume of 100–120 ml was harvested at passages 4–7. The processing and expansion of the cells took place at the Good Manufacturing Practice Stem Cell Laboratory Facility of Sun Yat-sen University, as previously described [19]. Characterization of the final product, which expressed CD90, CD73, CD105, CD44, and CD166, was determined by flow cytometric analysis. Before infusion, the UC-MSCs were subjected to aerobic, anaerobic, and fungal cultures and tested for mycoplasma infection; subsequently, their sterility was confirmed.

### Procedures

A stem-cell dose of 2 × 10^6^ UC-MSCs per kilogram body weight was administered for 30 min via the peripheral vein before renal transplantation. Vital signs were monitored continuously during the infusion. The dose of UC-MSCs administered via the renal artery during the surgical procedure was 5 × 10^6^ cells. Before releasing the renal artery, the targeted UC-MSCs were administered into the kidney and maintained for 10 min (Fig. 1).

### Clinical assessments and data collection

Participants were observed during the infusion and monitored for infusion reactions before operation. Additional adverse events (AEs) were identified through interviews with the participants during UC-MSC administration. For renal artery administration, renal perfusion after releasing the allograft artery was observed, and we assessed whether the renal capsule was full and plump and the renal cortex had ischemic areas. Additionally, urine volume from the release of the allograft vessels was also observed. After hospital discharge, AEs were identified through clinic visits or phone interviews with the participants at 3, 9, and 12 months after infusion.

Baseline clinical demographics of the donors (age, gender, body mass index, medical history, type of allograft, infection status, serum creatinine, causes of death, time of ICU, urine volume per day, creatinine level at organ procurement, time of warm and cold ischemia, vasoactive drugs, and cardiopulmonary resuscitation, CMV infection status) were documented. For the recipients, the clinical data included age, gender, medical history, current medication, blood type, previous blood transfusions, panel-reactive antibody (PRA), infection status, physical and laboratory examinations, and dialysis methods and duration. Other specifications, such as the number of human leukocyte antigen (HLA) mismatching between donor and recipient, complement dependent cytotoxicity, time-zero biopsy of the donated kidney, were also collected. All immunosuppressive agents and other drugs used and dosages administered were recorded during the study. The Modification of Diet in Renal Disease 2006 formulae were used to calculate the estimated glomerular filtration rate (eGFR) [20].

Serum creatinine and blood urea nitrogen levels, and urine volume per day were recorded to evaluate the renal function. Renal color ultrasound was performed postoperatively. In our study, DGF was defined as the use of dialysis in the first postoperative week, or failure of serum creatinine to decrease by 10% in the first 48 h following transplantation [21]. When acute rejection is suspected clinically, renal allograft biopsy was performed and classified according to Banff 2013 classification [22]. Incidences of pulmonary and urinary tract infections as well as other opportunistic infections such as CMV infection were monitored after the operation. Additionally, laboratory examinations, such as routine blood test, routine urine test, liver and renal function, and concentration of CNI, were performed once a week for 3 months after the operation and, thereafter, once every 2 weeks for 3–6 months and once a month for 6–12 months.

### Statistical methods

Differences in clinical characteristics of the participants were examined with paired t test for continuous variables and McNemar’s test for discrete variables. Kaplan–Meier curves were plotted to depict graft and recipient survivals, and a curve comparison was performed between the two groups using the log-rank test. All analyses were performed using the Statistical Package for Social Science 21.0 for Windows (IBM Corp., Armonk, NY). P value < 0.05 was considered statistically significant.

## Results

### Baseline characteristics of donors and recipients

Table 1 shows the qualitative and quantitative variables of the 21 donors. The mean age of the donors was 41.0 ± 11.5 years (> 40 years, 42.9%; men, 85.7%). The most frequent cause of death was head trauma (61.8%), followed by cerebrovascular accident (23.8%), and others (14.4%), and 33.3% of the donors had a history of hypertension and 4.8% had diabetes. In 85.7% of the donors, vasoactive drugs were used before organ procurement, and 14.3% of the donors received cardiopulmonary resuscitation. Terminal serum creatinine was 188.5 ± 113.9 μmol/l. The mean duration of warm ischemia time was 11.1 ± 4.5 min. No CMV infection was found in all donors.

**Table 1:** Demographics of the donors included in this study

Clinical values	N = 21
Age (years)	41.0 ± 11.5
Gender (% female)	14.3
Cause of death, n (%)	
Cerebral trauma	13 (61.8)
Cerebrovascular accident	5 (23.8)
Others	3 (14.4)
Using of vasoactive drugs, n (%)	18 (85.7)
Cardio-pulmonary resuscitation, n (%)	3 (14.3)
ICU time of donor (days)	4.6 ± 3.5
Donor BMI (kg/m^2^)	22.3 ± 2.7
Terminal donor Cr (μmol/L)	188.5 ± 113.9
History of arterial hypertension, n (%)	7 (33.3)
History of heart disease, n (%)	0
History of diabetes, n (%)	1 (4.8)
Warm ischemia time (min)	11.1 ± 4.5

*ICU* intensive care unit, *BMI* body mass index, *Cr* creatinine

Clinical variables of the 42 recipients were also described. The mean age of the recipients was 43.9 ± 10.1 years (59.5% were men, 97.5% were recipients of first transplants, and only one patient had a previous transplantation). The mean preoperative serum creatinine level was 1036.9 ± 290.2 μmol/l, with a mean dialysis duration of 2.2 ± 1.9 years. The mean BMI of recipients was 22.9 kg/m^2^ (range 18.5–30 kg/m^2^). None of the recipients had positive PRA, and 20 recipients had 0–1 of 6 possible mismatches at the HLA-A, HLA-B and HLA-DR loci. The mean duration of cold ischemia time was 5.2 ± 1.6 h. All transplants were ABO compatible.

The matched group where paired recipients received graft donations from the same donors was investigated (Table 2). Based on the use of UC-MSCs, 42 recipients who received graft donations from the same donors were divided into two groups: MSC group (n = 21) and non-MSC group (n = 21). No significant differences in baseline characteristics were found between the two groups (P > 0.05). 15 cases received tacrolimus in MSC-group, compared with 14 cases in no-MSC group, which has no significance in the two groups (P = 0.5) .

**Table 2:** Paired recipients who received graft donations from the same donors stratified by MSC

Recipient characteristics (paired)	Trials	Control	P value
Age (years)	40.8 ± 9.2	47.1 ± 10.2	0.60
Sex			
Male	14	11	0.53
Female	7	10	
Preoperative Cr (μmol/L)	1106.4 ± 326.9	967.3 ± 236.0	0.28
Previous transplants			
First transplant	20	21	1
Second transplant	1	0	
HLA mismatches			
Level 1	11	9	0.76
Level 2	10	12	
Pre-emptive dialysis vintage (years)	2.4 ± 2.2	2.1 ± 1.6	1
Cold ischemia time (h)			
< 6	14	13	1
6–12	7	8	
Postoperative complications			
DGF	2	7	0.13
AR	3	1	0.61
Complicated urinary tract infection	1	1	1
Severe pneumonia	2	5	0.41
Severe bleeding	0	0	1
Renal allograft resection or embolism	1	1	1
Other	1	1	1

*MSC* mesenchymal stem cell, *Cr* creatinine, *HLA* human leukocyte antigen, *DGF* delayed graft function, *AR* acute rejection

For sex-mismatch transplants, six female recipients of male allografts, and one male recipient of female allograft were found in MSC group compared with nine female recipients of male allografts, and one male recipient of female allograft in no-MSC group (P = 0.67).

### Postoperative complications and safety monitoring

No significant difference in postoperative complications between the two groups was noted (all P > 0.05; Table 2**)**. In the MSC group, 9.5% developed DGF 1 week postoperatively; in the non-MSC group, 33.3% (P = 0.13). Moreover, 14.3% of cases from the MSC group and 4.8% in non-MSC group had AR (P = 0.61). Although the incidences of severe infections related to urinary and respiratory tracts were lower in the MSC group, no significant difference was found (P = 0.46). No CMV infection occurred in all patients.

All patients tolerated the MSC infusion, and no adverse clinical effects, such as fever, headache, vomiting, weakness, hematuria, or allergic reactions, were observed. AEs related to cell infusion were not detected in any of the patients during the 12-month follow-up.

### Kidney function outcomes

**Fig. 2:**
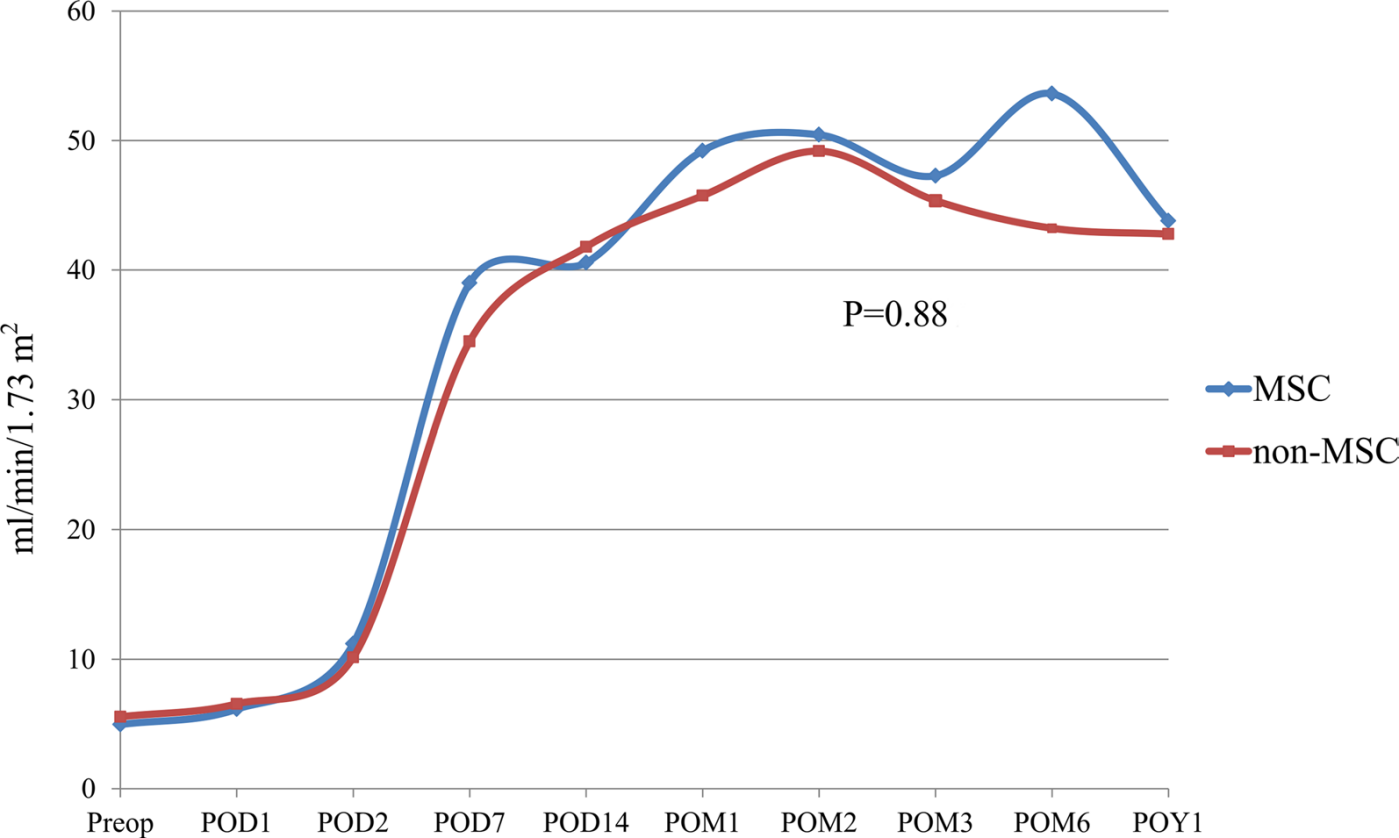
The eGFR curves at different time points during the follow-up period in UC-MSCs and non-MSC groups. No significant difference in eGFR changes postoperatively between the two groups was found (P = 0.88)

Serum creatinine levels were recorded at different follow-up time points, and no significant difference in serum creatinine curves was found between the two groups (P > 0.05). For the eGFR, patients in the MSC group had a mean eGFR value of 4.95 ± 1.73 ml/min/1.73 m^2^ at baseline, which increased to 43.80 ± 16.21 ml/min/1.73 m^2^ at the 12-month visit. The mean eGFR of patients in the non-MSC group increased from 5.48 ± 1.50 ml/min/1.73 m^2^ (baseline) to 42.78 ± 23.15 ml/min/1.73 m^2^ 1 year after cell infusion (P = 0.88) (Fig. 2).

### Graft and patient outcomes following kidney transplantation

**Fig. 3:**
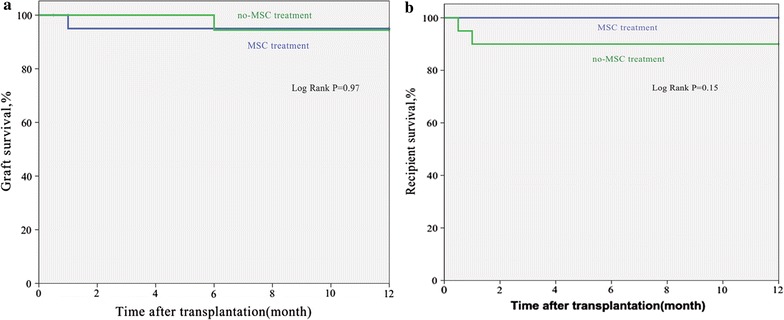
Kaplan–Meier survival estimates after renal transplantation in recipients of kidneys with or without UC-MSCs. **a** Graft survival in the MSC group was comparable to that in the non-MSC group (P = 0.97). **b** A similar analysis revealed no significant difference in recipient survival between the two groups (P = 0.15)

The median follow-up time for graft and recipient in the MSC and non-MSC groups was 12 months (range 1–12 months, and 12 months for graft and recipient in MSC group; 0.5–12 months in non-MSC group). Graft survival in the MSC group was comparable to that in the non-MSC group (P = 0.97; Fig. 3a). A similar analysis revealed no significant difference in recipient survival between the two groups (P = 0.15; Fig. 3b). One-year graft and recipient survivals were comparable between the MSC and non-MSC groups (95.2 versus 95.2%, P = 0.76 and 100 versus 90.5%, P = 0.24, respectively). In the MSC group, one patient had renal allograft ruptures postoperatively due to peri-renal abscess and resected transplanted kidneys. In the non-MSC group, one patient had renal allograft embolism. Two patients in the non-MSC group died of severe pneumonia.

### Location of MSCs in vivo

**Fig. 4:**
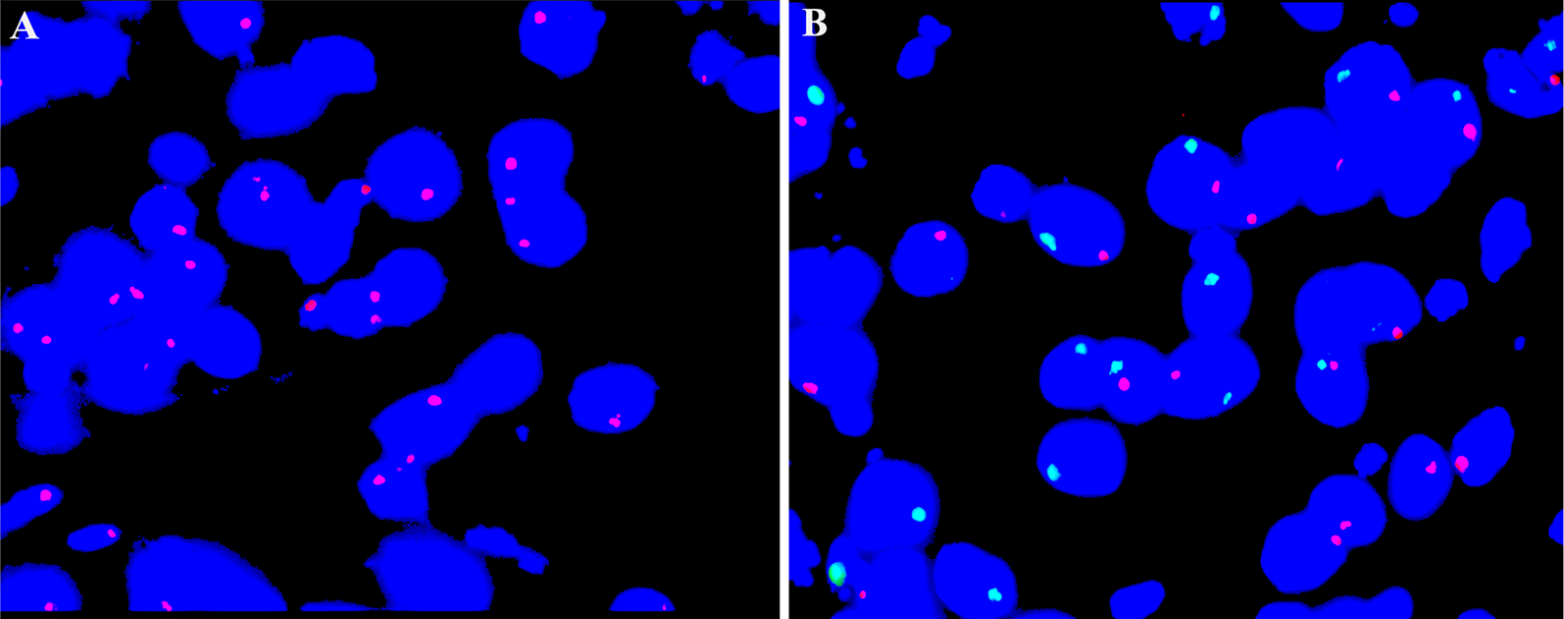
Detection of UC-MSCs in a recipient’s biopsy sample by a multiprobe FISH assay. No UC-MSCs with chromosomes “XY” were found in a female recipient’s biopsy sample. **A** The female recipient’s biopsy sample showed two red signals representing chromosomes “XX”. Original magnification of FISH images, oil objective (×100). **B** The FISH assay showed one red and one green signals representing chromosomes “XY” in a control male recipient’s biopsy sample. Original magnification of FISH images, oil objective (×100)

To understand the effect of MSCs on the renal allograft, we performed a multiprobe fluorescence in situ hybridization (FISH) assay to detect cell location using a kidney biopsy sample. We selected a dose of UC-MSCs, which were isolated after birth with written consent from the parents. Chromosomes “XY” were confirmed by the multiprobe FISH assay. Subsequently, the selected UC-MSCs were administered to female recipients according to the aforementioned process. Based on the protocol, biopsy of the renal allograft was performed 7 days after the operation. Multiprobe FISH assay was also conducted to detect whether UC-MSCs are present in the biopsy sample. No UC-MSCs with chromosomes “XY” were found in the female recipient’s biopsy sample (Fig. 4A, B).

## Discussion

To the best of our knowledge, this is the first multicenter randomized paired trial that showed the safety and feasibility of UC-MSCs infusion in patients undergoing kidney transplantation. We found that systemic administration of fresh UC-MSCs as induction therapy could achieve a substantial reduction in the incidence of DGF, although no significant difference was found between the MSC and non-MSC groups. Additionally, we also demonstrated that the administration of UC-MSCs via the renal artery during operation is not feasible because no injected MSCs were observed in the renal allograft.

Mesenchymal stem cells not only can limit inflammatory responses but also have the potential to induce antifibrotic activity and tissue regeneration [23–25]. Multiple studies using animal models have attempted to prove the beneficial effects of MSC on tissue injury and inflammation and presented promising results; however, few studies that assess the safety and efficacy of MSCs clinically have been conducted. In a recent study, patients administered with autologous MSCs had a lower incidence of acute rejection and opportunistic infection and better eGFR at 1 year postoperatively [12]. Although recent MSC trials in renal transplantation presented evidence on the benefits of MSC, no studies on the simultaneous prevention of DGF and acute rejection by MSCs in deceased donor renal transplantation exist. Additionally, most studies used autologous MSCs, which possess several key disadvantages due to lack of donor selection and availability “off-the-shelf” for clinical use without the delay required for expansion [26]. Moreover, numerous different challenges in the use of MSCs in clinical trials exist. Firstly, because of the absence of updated and adequate good manufacturing practice guidelines for MSC-based therapy for the kidneys, MSCs may be derived from different materials, which may in turn exert a different influence on clinical trial results [27]. Secondly, no studies involving a large dose of MSCs have been conducted, and in most studies, the dose and frequency of MSC administration are empirically chosen. Studies on renal transplantation typically involved a dose ranging from 0.58 × 10^6^ to 5.0 × 10^6^ MSCs per kilogram body weight; however, in graft-versus-host disease, a higher dose of 9.0 × 10^6^ MSCs was administered [25]. The route of MSC administration is another aspect that should be considered in renal treatment. In most clinical studies, intravenous infusion administration was performed; however, cells administered intravenously could also be attracted to lung tissues, thereby affecting the accurate homing of the cells to the renal tissues [28]. Hence, specific delivery of MCSs needs to be performed. It must be noted that MSC-based therapy is currently being developed. Nevertheless, this therapy would be considered a new approach for the treatment of kidney disease. To obtain better outcomes, randomized and controlled multicenter clinical trials are necessary [29].

Consequently, we performed this multicenter randomized paired trial of MSCs to clarify the clinical value of UC-MSCs as induction therapy to prevent both DGF and acute rejection in deceased donor grafts. The ultimate goal of this approach was to achieve low DGF and acute rejection incidences as well as long-term transplant survival. Moreover, confirming the safety and efficacy of human UC-MSCs could expand the source of MSCs. Potential risks of MSCs in solid organ transplantation include direct toxicity, malignancies, and risks for excessive immunosuppression and immunogenicity [30, 31]. In previous studies, some opportunistic infections including BKV nephropathy and cytomegalovirus infections were observed because of immunosuppression related to MSCs [12, 13]. Additionally, due to a short follow-up period and the inclusion of ill patients with poor prognosis, it was difficult to find an association between new malignancies and the use of MSC therapy [32]. In our study, MSCs infusion was safe and well tolerated during the perioperative and follow-up periods with no adverse events, which could be attributed to the adequate good manufacturing practice guideline for MSCs in our center. Moreover, the single dose of 2 × 10^6^ UC-MSCs per kilogram body weight was insufficient to induce direct toxicity and excessive immune suppression and immunogenicity. Risk of malignancies could be evaluated with a longer follow-up period.

In our study, administration of UC-MSCs could not decrease the incidence of DGF and acute rejection. We speculate that the timing and frequency of UC-MSC administration limited the effect on the renal allografts. The timing of MSC administration has been studied in some ischemic disease models [33, 34]. In a previous study, MSCs used immediately or 24 h post ischemia/reperfusion (I/R) induced significant renoprotection [35]. In a sheep model, MSCs treatment for renal I/R showed that treatment with MSCs at 15 days post-I/R was inferior to immediate treatment [36]. A recent meta-analysis also revealed that MSCs administration 1 day after injury yielded greater therapeutic value than that within 24 h of injury [37]. In our study, three disadvantageous processes possibly limited the positive effect of UC-MSCs administered before the operation. First, the inflammation environment in the early injured kidney was unfavorable for MSCs survival within the tissue, thereby decreasing the stem cell effect. Second, the insufficient expression of the homing adhesion molecules ICAM-1 and VCAM-1 prevented MSCs integration into the injured kidney tissue [38]. Third, upregulated pro-inflammatory markers in the I/R kidney tissue, such as TNF-α and IL-1β, prevented the effect of MSCs.

Moreover, we performed a multiprobe FISH assay to detect cell location in a kidney biopsy sample and showed that no UC-MSCs were found in the female recipient’s biopsy sample, which is consistent with aforementioned explanations on the effects of MSCs administration. Additionally, only one dose of UC-MSCs was administered during the operation, which was possibly insufficient to have an effect on renal allograft outcome. Hence, several infusions may be more preferable than one infusion [10]. In a previous clinical trial, a high dose, ranging from 150 to 300 million MSCs, was used because of the low survival and engraftment of MSCs after transplantation. However, this could result in transfusion reactions such as allergic reaction, fever, hypotension, and infection [39]. Thus, we suggested that increasing MSCs administration frequency, such as twice per week over the course of 2 weeks, may provide a sufficient number of cells and prevent risks of transfusion reaction.

In our study, graft and recipient survivals and the incidence of postoperative complications were comparable between the two groups. Postoperative renal function recovery could be understood through changes in glomerular filtration rate (GFR) curves. We compared the GFR curves postoperatively between the MSC and non-MSC groups; no significant difference was found, indicating similar renal function recovery between the groups.

Our results have some promising significance for future work with UC-MSCs therapy in renal transplantation. However, several limitations of this study must be considered. Firstly, information about the long-term outcome of UC-MSCs therapy in renal transplantation was not available in our study because of the limited follow-up time. Secondly, we failed to fully demonstrate the efficacy of the treatment due to the small sample size and the single dose of cell infusion. Moreover, the small sample size in this study makes full assessment of the differences in DGF incidence difficult, although a lower incidence was found in the MSCs group.

## Conclusions

Mesenchymal stem cell-based cell therapy to prevent complications associated with renal transplantation is considered a new horizon. Our pilot study suggests that adequate administration timing and frequency of UC-MSCs possibly have an important effect on graft and recipient outcomes. Furthermore, direct administration of UC-MSCs via the renal artery is insufficient because of the observed absence of injected MSCs in renal tissues. Nevertheless, it must be noted that the therapy is still at the preliminary stages of development. Further studies are needed to fully establish the administration of MSCs as a novel induction therapy in renal transplantation.

## Abbreviations

DGF: delayed graft function
MSCs: mesenchymal stem cells
UC-MSCs: umbilical-cord-derived MSCs
WHO: World Health Organization
HIV: human immunodeficiency virus
AE: adverse events
ICU: intensive care unit
CMV: cytomegalovirus
PRA: panel-reactive antibody
HLA: human leukocyte antigen
eGFR: estimated glomerular filtration rate
CNI: calcineurin inhibitor
FISH: fluorescence in situ hybridization
BKV: BK virus
I/R: ischemia/reperfusion
ICAM-1: intercellular cell adhesion molecule-1
VCAM-1: vascular cell adhesion molecule-1
GFR: glomerular filtration rate
